# Role of protein kinase C and epidermal growth factor receptor signalling in growth stimulation by neurotensin in colon carcinoma cells

**DOI:** 10.1186/1471-2407-11-421

**Published:** 2011-10-02

**Authors:** Kristin M Müller, Ingun H Tveteraas, Monica Aasrum, John Ødegård, Mona Dawood, Olav Dajani, Thoralf Christoffersen, Dagny L Sandnes

**Affiliations:** 1 Department of Pharmacology, Institute of Clinical Medicine, Faculty of Medicine and Oslo University Hospital, University of Oslo, Oslo, Norway; 2 Department of Oncology, Oslo University Hospital, Oslo, Norway

## Abstract

**Background:**

Neurotensin has been found to promote colon carcinogenesis in rats and mice, and proliferation of human colon carcinoma cell lines, but the mechanisms involved are not clear. We have examined signalling pathways activated by neurotensin in colorectal and pancreatic carcinoma cells.

**Methods:**

Colon carcinoma cell lines HCT116 and HT29 and pancreatic adenocarcinoma cell line Panc-1 were cultured and stimulated with neurotensin or epidermal growth factor (EGF). DNA synthesis was determined by incorporation of radiolabelled thymidine into DNA. Levels and phosphorylation of proteins in signalling pathways were assessed by Western blotting.

**Results:**

Neurotensin stimulated the phosphorylation of both extracellular signal-regulated kinase (ERK) and Akt in all three cell lines, but apparently did so through different pathways. In Panc-1 cells, neurotensin-induced phosphorylation of ERK, but not Akt, was dependent on protein kinase C (PKC), whereas an inhibitor of the β-isoform of phosphoinositide 3-kinase (PI3K), TGX221, abolished neurotensin-induced Akt phosphorylation in these cells, and there was no evidence of EGF receptor (EGFR) transactivation. In HT29 cells, in contrast, the EGFR tyrosine kinase inhibitor gefitinib blocked neurotensin-stimulated phosphorylation of both ERK and Akt, indicating transactivation of EGFR, independently of PKC. In HCT116 cells, neurotensin induced both a PKC-dependent phosphorylation of ERK and a metalloproteinase-mediated transactivation of EGFR that was associated with a gefitinib-sensitive phosphorylation of the downstream adaptor protein Shc. The activation of Akt was also inhibited by gefitinib, but only partly, suggesting a mechanism in addition to EGFR transactivation. Inhibition of PKC blocked neurotensin-induced DNA synthesis in HCT116 cells.

**Conclusions:**

While acting predominantly through PKC in Panc-1 cells and via EGFR transactivation in HT29 cells, neurotensin used both these pathways in HCT116 cells. In these cells, neurotensin-induced activation of ERK and stimulation of DNA synthesis was PKC-dependent, whereas activation of the PI3K/Akt pathway was mediated by stimulation of metalloproteinases and subsequent transactivation of the EGFR. Thus, the data show that the signalling mechanisms mediating the effects of neurotensin involve multiple pathways and are cell-dependent.

## Background

Due to the high prevalence of colorectal cancer [1], better insight into regulatory mechanisms involved in cell proliferation in this malignancy is needed, and might ultimately lead to improved treatment. Several receptors can mediate proliferogenic signals. Among these, G protein-coupled receptors (GPCRs) may induce mitogenic signalling and have a role in cancer, including colorectal and pancreatic cancer [2-4]. Moreover, activation of GPCRs and receptor tyrosine kinases (RTKs) may act in concert to enhance cellular proliferation. Thus, an important question is how these signals are integrated in the cells.

GPCRs are heptahelical transmembrane receptors mediating their effects via heterotrimeric G proteins (of either the Gs, Gi, Gq, or G12/13 subtypes) [5,6]. While the role of Gs-coupled prostanoid receptors in colon cancer cell proliferation, apoptosis, and migration has been extensively studied [4], there is less information on the role of Gq-coupled receptors in this malignancy. Stimulation of these receptors leads to activation of phospholipase Cβ (PLCβ) and thereby of protein kinase C (PKC), which may be involved in tumorigenesis [7]. Elevated expression of PKC βII has been found to be an early promotive event in colon cancer development [8], and inhibition of PKC β was found to decrease proliferation and induce apoptosis in colon carcinoma cells [9].

Neurotensin is a peptide that binds to GPCRs. It is mainly formed in the central nervous system and by endocrine cells of the digestive tract, where it acts as a paracrine and endocrine modulator in a variety of gut functions, including vascular smooth muscle activity, gastrointestinal motility, gastric emptying, and intestinal, pancreatic, and biliary secretions [10]. In addition, neurotensin stimulates growth of the intestinal mucosa under physiological and pathological conditions [10,11] and has been found to promote azoxymethane-induced colon carcinogenesis in rats and mice [12,13]. Neurotensin has also been implicated in the progression of cancers of the pancreas, breast, lung, and prostate [10,11,14]. Three subtypes of neurotensin receptors have been cloned [15]. The high affinity NTSR1 receptor and the low affinity NTSR2 receptor both belong to the GPCR family, while the NTSR3/sortilin receptor is a nonspecific receptor with a single transmembrane domain [15,16]. The pharmacological and signalling properties of the NTSR2 receptor, which exerts its effects mainly in the central nervous system, are incompletely understood, and appear to be dependent on cell type and species [16]. The peripheral effects of neurotensin appear to be mediated largely by NTSR1, which activates PLCβ [14,16]. Experiments using a specific antagonist or knockdown of the NTSR1 using short interfering RNA suggest that NTSR1 mediates the effects of neurotensin on cancer cells, although NTSR3/sortilin, which is often coexpressed in cancer cells, may modulate NTSR1 signalling [14,16]. Splice variants of the NTSR1 were recently detected in prostate cancer cell lines, however, no functional studies of these have been conducted [17]. Recent data have suggested that the NTSR1 receptor gene may be a downstream target of the extracellular signal-regulated kinase (ERK) and Tcf/β-catenin pathways [18,19], and increased expression of NTSR1 during progression of colon tumorigenesis has been reported [20,21].

Neurotensin has been found to stimulate proliferation of certain colon carcinoma cell lines [10,22]. Reports on intracellular signalling leading to proliferation induced by neurotensin in some other cell types have suggested the involvement of PKC-dependent activation of ERK and protein kinase D (PKD) [10,23-27], and either dependence or independence of epidermal growth factor receptor (EGFR) transactivation [23-25]. In the pancreatic cancer cell line Panc-1, DNA synthesis induced by neurotensin was independent of EGFR transactivation [23], whereas in the prostate cancer cell line PC-3, neurotensin stimulated mitogenesis by a PKC-dependent transactivation of EGFR [24]. In colon carcinoma cell lines neurotensin has been found to activate ERK, as well as PKC, Akt, and nuclear factor κB (NF-κB) pathways [28-31]. Furthermore, neurotensin induced phosphorylation and inactivation of glycogen synthase kinase (GSK), leading to cyclin D1 expression, through mechanisms that were at least partly dependent on PKC [29]. Neurotensin has also been found to induce a proinflammatory tumour microenvironment and promote cancer cell invasion through pathways that involved NF-κB, PKC, ERK, and the sodium-proton exchanger 1 (NHE1) [14,31-33].

The aim of the present study was to investigate some of the intracellular signalling pathways involved in mitogenesis induced by neurotensin in human colorectal cancer cells, by examining the HCT116 and HT29 lines and comparing them with Panc-1 cells. The results suggested that while neurotensin acted predominantly through PKC in Panc-1 cells and via EGFR transactivation in HT29 cells, it used both these pathways in HCT116 cells. In the latter cells neurotensin-induced activation of ERK was mediated largely by PKC, while neurotensin-induced activation of Akt was independent of PKC but involved transactivation of the EGFR, apparently by a Ca^2+^-dependent mechanism. Neurotensin-induced DNA synthesis was mediated mainly by PKC.

## Methods

### Chemicals

Dulbecco's modified Eagle's medium, N-(2-hydroxyethyl)piperazine-N'-(2-ethanesulfonic acid (Hepes), penicillin and streptomycin were from Gibco (Grand Island, NY). Neurotensin, 12-O-tetradecanoylphorbol-13-acetate (TPA), thapsigargin, epidermal growth factor (EGF), and wortmannin were obtained from Sigma-Aldrich (St. Louis, MO). [2-[1-(3-dimetylaminopropyl)-1*H*-indol-3-yl]-maleimide] (GF109203X), 4-(3-chloroanilino)-6,7-dimethoxyquinazoline (tyrphostin AG1478), 2'-amino-3'-methoxyflavone (PD98059, and N-[(2R)-2 (hydroxamidocarbonylmethyl)-4-methylpentanoyl]-L-tryptophan methylamide (GM6001/Galardin) were from Calbiochem (San Diego, CA). 7-Methyl-2-(4-morpholinyl)-9-[1-(phenylamino)ethyl]-4H-pyrido[1,2-a] pyrimidin-4-one (TGX-221) was obtained from Cayman Chemical (Ann Arbor, MI). Transforming growth factor α (TGFα) was obtained from Bachem (Bubendorf, Switzerland). 4-Quinazolinamine, N-(3-chloro-4-fluorophenyl)-7-methoxy-6-[3-4-morpholin)propoxy] (gefitinib) was a gift from Astra Zeneca (Cheshire, UK), and cetuximab was kindly provided by Merck KgaA (Darmstadt, Germany). [6-^3^H]thymidine (20-30 Ci/mmol) and *myo*-[2-^3^H]inositol (15.0 Ci/mmol) were from Amersham Biosciences (Buckinghamshire, UK). Antibodies against phosphorylated Akt^Ser473^, total Akt, dually phosphorylated ERK^Thr202/Tyr204^, phospho-EGF receptor^Tyr1173^, and phospho-Shc ^Tyr239/240 ^were obtained from Cell Signaling Technology (Boston, MA). Anti-ERK and anti-Shc antibodies were obtained from Upstate (Billerica, MA). EGFR antibody (1005) was obtained from Santa Cruz Biotechnology, Inc. (Santa Cruz, CA). Secondary antibodies were purchased from Bio-Rad Laboratories (Hercules, CA) and Licor Biosciences (Lincoln, NE). All other chemicals were of analytical quality. Stock solutions of test compounds were prepared in DMSO (TPA, thapsigargin, wortmannin, PD98059, GM6001, TGX221, gefitinib) or 0.9% NaCl (neurotensin, GF109203X). EGF was dissolved in 4 mM HCl, and TGFα in 4 mM HCl containing 1% bovine serum albumin from Sigma Aldrich (St. Louis, MO). Cetuximab was dissolved in phosphate-buffered saline (PBS). When solutions containing DMSO were used, the final concentration of DMSO was kept as low as possible.

### Cell culture

Human colorectal cancer cell lines HCT116 and HT29, and pancreatic adenocarcinoma cell line Panc-1 were obtained from ATCC (Manassas, VA). The cells were maintained in Dulbecco's modified Eagle's medium containing 1 g/l glucose (or 4.5 g/l for Panc-1) supplemented with 10% horse serum (10% fetal bovine serum for Panc-1 cells), penicillin (67 μg/ml), streptomycin (100 μg/ml) and 2 mM glutamine (4 mM for Panc-1). Cells were plated onto Costar plastic culture wells (Corning Life Sciences, Acton, MA) at a density of 50 000 cells/cm^2 ^(25 000 cells/cm^2 ^in the case of Panc-1 cells) in serum-containing medium. The cultures were kept in 95% air/5% CO_2 _at 37°C. After 24 hours the medium was replaced with serum-free medium and the cells were cultured for 24 hours before stimulation with agonists.

### Measurement of DNA synthesis

Neurotensin, TPA, and inhibitors of PKC and EGF receptor were added to serum-starved HCT116 cells as described in the figure legends, and [^3^H]thymidine was added 12 hours after stimulation. Serum-starved HT29 and Panc-1 cells were stimulated for 21 hours with neurotensin and EGF before [^3^H]thymidine was added. The cells were harvested after three hours pulsing with [^3^H]thymidine, and DNA synthesis was measured as the amount of radioactivity incorporated into DNA as previously described [34]. Briefly, medium was removed, and cells were washed twice with 0.9% NaCl. The cellular material was dissolved with 1.5 ml of 0.5 N NaOH for 3 hours at 37°C, collected, mixed with 1.5 ml H_2_O, and precipitated with 0.75 ml 50% trichloroacetic acid (TCA). The acid-precipitable material was transferred to glass fiber filters (GF/C Whatman, GE Healthcare, UK) and washed twice with 5.0 ml 5% TCA, followed by liquid scintillation counting of the filters in a Packard Tri-Carb liquid scintillation counter.

### Inositol phosphate accumulation

Cells were labelled with [^3^H]inositol, 2.5 μCi/ml for 24 hours in serum-free medium. Medium was removed 30 minutes before agonist stimulation and replaced with Krebs-Ringer-Hepes buffer pH 7.4, containing 10 mM glucose and 15 mM LiCI. HCT116 cells were stimulated with neurotensin for 30 minutes, and the reaction was stopped by removing buffer and adding 1 ml ice-cold 0.4 M perchloric acid. Samples were harvested and neutralized with 1.5 M KOH, 60 mM EDTA, 60 mM Hepes, in the presence of Universal indicator. The neutralized supernatants were applied on columns containing 1 ml Dowex AG 1-X8 resin (Bio-Rad Laboratories, Hercules, CA), and inositol phosphates were eluted with 10 ml 1 M ammonium formate/0.1 M formic acid.

### Immunoblotting

Aliquots with ~30 000 cells (total cell lysate prepared in Laemmli or RIPA buffer) were electrophoresed on 6-12% (w/v) polyacrylamide gels. This was followed by protein electrotransfer to nitrocellulose membranes and immunoblotting with antibodies against phospho-Akt, total Akt, phospho-ERK1/2, total ERK, phospho-EGFR, total EGFR, phospho-Shc, and total Shc, respectively. Immunoreactive bands were visualized with enhanced chemiluminescence using LumiGLO (KPL Protein Research Products, Gaithersburg, MD), or infrared imaging using Odyssey Infrared Imaging System supplied by Licor Biosciences (Lincoln, NE), respectively.

### Statistical analyses

Results are expressed as means ± standard error of the mean (S.E.M). DNA synthesis data were analyzed by one-way ANOVA, and post tests using Bonferroni correction to compare groups, using GraphPad Prism (version 5.01, GraphPad Software, San Diego, California, USA). Results were considered significant when p < 0.05.

## Results

### Neurotensin stimulates DNA synthesis in HCT116 and Panc-1 cells

**Figure 1:**
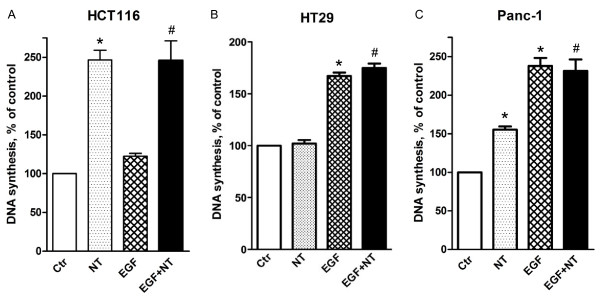
**Effect of EGF and neurotensin alone and in combination on DNA synthesis**. Quiescent HCT116 (A), HT29 (B), and Panc-1 (C) cells were treated with 5 nM EGF, 1 μM neurotensin (NT), or a combination of both agents. [3 H]thymidine was added 12 h (HCT116) or 21 h after addition of agonists. The cells were harvested three hours after adding [3 H]thymidine and DNA synthesis was assessed as described in Methods. The results are presented as per cent of control values of three (HCT116) or six (HT29, Panc-1) independent experiments. Error bars indicate S.E.M. * Significantly different (p < 0.05) from control; # significantly different from control (p < 0.05), but not from neurotensin alone (A) or EGF alone (B, C).

Neurotensin has been reported to act as a mitogen in certain colon cell lines [10,22]. We found that neurotensin dose-dependently induced DNA synthesis in HCT116 cells, reaching a two- to three-fold increase as compared to basal levels (Figure 1A, 2C). In contrast, addition of EGF only slightly increased DNA synthesis, which is in agreement with previous data and might be explained by an autocrine production of EGFR ligands by these cells, masking the effects of exogenously added EGF [35-37]. Furthermore, concomitant stimulation of HCT116 cells with neurotensin and EGF did not induce any synergistic or additive effect on DNA synthesis. In HT29 cells, EGF dose-dependently (data not shown) stimulated DNA synthesis, whereas neurotensin had no significant effects, neither alone nor in combination with EGF (Figure 1B). In Panc-1 cells, both neurotensin and EGF stimulated DNA synthesis, as reported previously [25], (Figure 1C).

**Figure 2:**
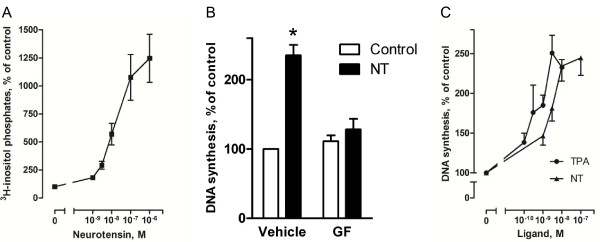
**Role of PKC in neurotensin-stimulated DNA synthesis in HCT116 cells**. A, Dose-response curve for the effect of neurotensin (NT) on accumulation of [3 H] inositol phosphates in the presence of 15 mM LiCl. Cells were labeled with [3 H]-inositol for 24 hours before NT was added to quiescent cells at the concentrations indicated. The reaction was stopped after 30 minutes, and inositol phosphates were extracted and analyzed as described in Methods. Results are presented as per cent of control values ± S.E.M., n = 3. B, Effect of the PKC inhibitor, GF109203X (GF) on basal and NT-induced DNA synthesis. Quiescent cells were pretreated with 3.5 μM GF109203X for 30 min before stimulation with 1 μM NT. Based on dose-response curves for the effect of GF109203X on NT-induced DNA synthesis we found 3.5 μM to be the maximum concentration tolerated by our cells (data not shown). DNA synthesis was determined by [3 H]thymidine incorporation as described in the legend of figure 1. Results are presented as per cent of control values of seven independent experiments. Error bars indicate S.E.M. * Significantly different from control (p < 0.05). C, Dose-response curves for the effects of NT or TPA on DNA synthesis. Quiescent cells were treated with the respective agonists in the concentrations indicated. DNA synthesis was determined by [3 H]thymidine incorporation as described in the legend of figure 1. The results are presented as per cent of control values ± S.E.M. of six independent experiments.

### Role of PKC in neurotensin-induced DNA synthesis

The high affinity NTSR1 receptor is known to activate PLC [15]. Neurotensin was previously shown to elevate intracellular Ca^2+ ^in HCT116 cells [38], and in our experiments neurotensin strongly and dose-dependently stimulated accumulation of inositol phosphates in these cells (Figure 2A). This strongly implicates PLC in the mechanisms of the cellular response of HCT116 cells to neurotensin. We next pretreated HCT116 cells with the PKC inhibitor GF109203X, and Figure 2B shows that this blocker strongly reduced DNA synthesis. It was also noted that the stimulatory effect of neurotensin on DNA synthesis was of the same magnitude as the effect of the direct PKC activator tetradecanoylphorbol acetate (TPA) (Figure 2C). Together, the results suggest a major role of the PLC/PKC pathway in the stimulation of DNA synthesis by neurotensin in these colon cancer cells.

### Role of PKC in neurotensin-induced phosphorylation of ERK

**Figure 3:**
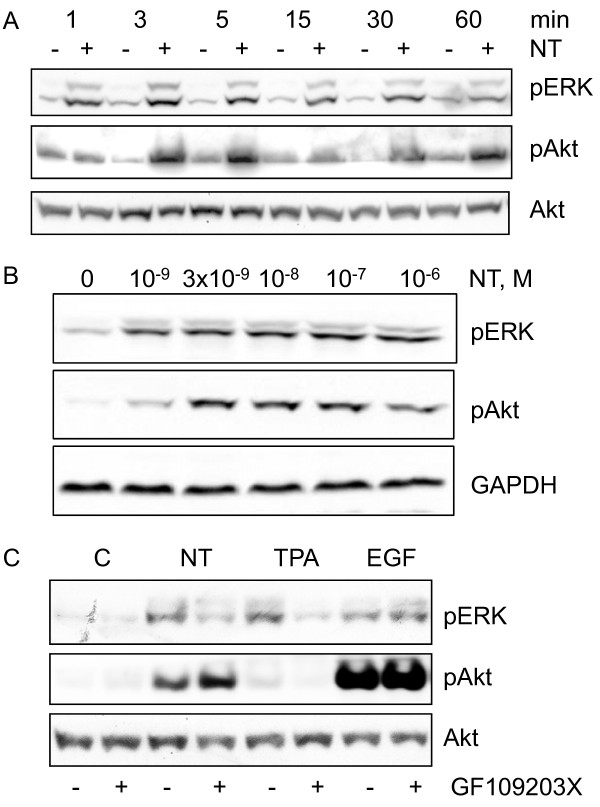
**Time-course and PKC-dependency of ERK and Akt phosphorylation in HCT116 cells**. A, Time-course of neurotensin-induced phosphorylation of ERK and Akt. Quiescent cells were stimulated with 1 μM neurotensin (NT) for the times indicated. Cells were harvested for subsequent Western analysis as described in Methods. Results represent one typical of three independent experiments. B, Effect of increasing concentrations of neurotensin on phosphorylation of ERK and Akt. Quiescent cells were stimulated with increasing concentrations of neurotensin for five minutes. Cells were harvested for subsequent Western analysis as described in Methods. Results represent one typical of three experiments. C, Role of PKC in NT-induced phosphorylation of ERK and Akt. Quiescent cells were pretreated with vehicle (0.9% NaCl) or 3.5 μM GF109203X for 30 min, before stimulation with 1 μM NT, 1 μM TPA, or 5 nM EGF for five min and then harvested for subsequent Western analysis. Results represent one typical of eight independent experiments.

**Figure 4:**
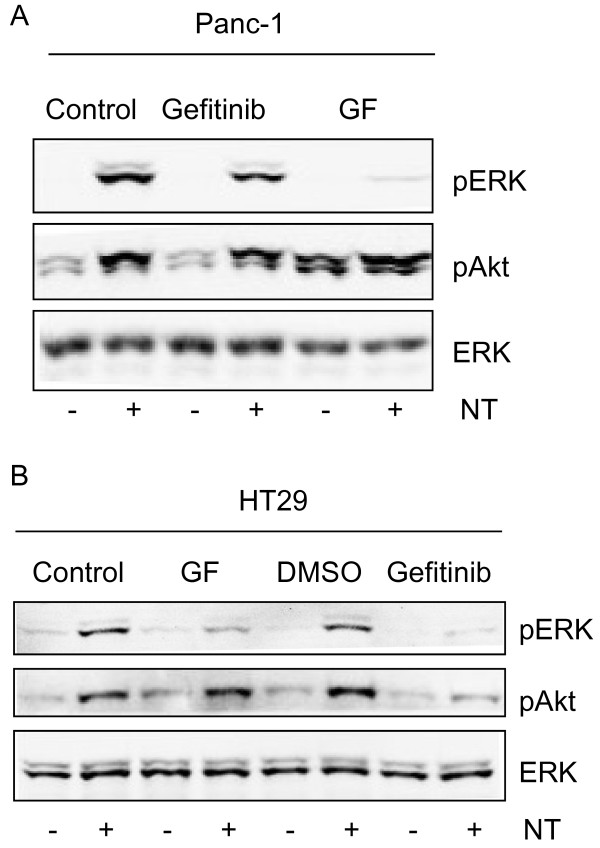
**Role of PKC and EGF receptor in neurotensin-induced signalling in HT29 and Panc-1 cells**. Panc-1(A) and HT29 (B) cells were pretreated with vehicle (0.9% NaCl or 0.5% DMSO), 3.5 μM GF109203X, or 1 μM gefitinib for 30 min before stimulation with 1 μM neurotensin (NT) for 5 min. Cells were harvested for subsequent Western analysis as described in Methods. Results represent one typical of four independent experiments.

Neurotensin induced a marked, rapid, and sustained phosphorylation of ERK in HCT116 cells (Figure 3A), which appeared to plateau at a concentration of 3-10 nM (Figure 3B). Direct activation of PKC by TPA also stimulated ERK phosphorylation (Figure 3C). The phosphorylation of ERK in response to neurotensin and TPA was strongly reduced by pretreatment of the cells with GF109203X (Figure 3C). In contrast, EGF-stimulated ERK phosphorylation was not affected by the PKC blocker (Figure 3C). In agreement with previous data [23] neurotensin stimulated ERK phosphorylation in a PKC-dependent manner in Panc-1 cells (Figure 4A), whereas in HT29 cells, ERK phosphorylation was only slightly attenuated by the PKC inhibitor (Figure 4B). Thus, in agreement with previous results from other cells where neurotensin stimulated ERK phosphorylation and DNA synthesis in a PKC-dependent manner [23-25], our data indicate that neurotensin-induced ERK phosphorylation in HCT116 cells is PKC-dependent.

### Role of EGFR in Akt phosphorylation induced by neurotensin

EGF induced a marked phosphorylation of Akt in HCT116 cells, indicating activation of the phosphoinositide 3-kinase (PI3K) pathway (Figure 3C). Neurotensin also stimulated phosphorylation of Akt, although not as strongly as EGF (Figure 3C). The effect of neurotensin on Akt first appeared after 3 min, while ERK phosphorylation was evident already at 1 min (Figure 3A). Furthermore, unlike the data indicating a PKC-mediated activation of ERK, neurotensin-induced phosphorylation of Akt was not affected by inhibition of PKC and was not mimicked by TPA (Figure 3C).

**Figure 5:**
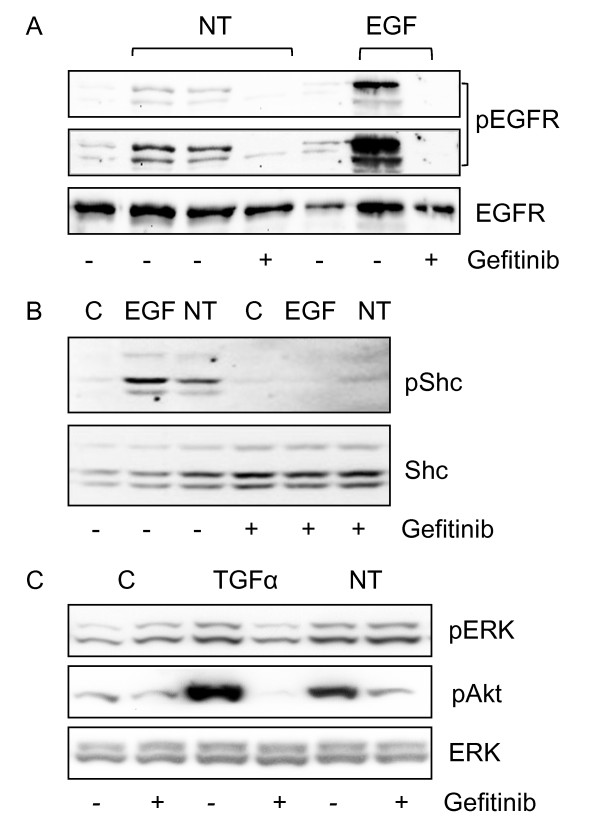
**Role of EGFR in neurotensin-induced signalling in HCT116 cells**. A, Phosphorylation of the EGFR residue 1173 induced by neurotensin (NT) and EGF. Quiescent cells were pretreated with vehicle (0.5% DMSO) or 10 μM gefitinib for 30 min before stimulation with 1 μM NT or 5 nM EGF for five min. Cells were harvested for subsequent Western analysis as described in Methods. Because of the strong EGFR phosphorylation induced by EGF, we also show the same blot overexposed. Results represent one typical of three independent experiments. B, Phosphorylation of Shc induced by NT and EGF in cells pretreated with vehicle or gefitinib. Results represent one typical of three independent experiments. C, Phosphorylation of ERK and Akt induced by NT (1 μM) and TGFα (10 nM) in cells pretreated with vehicle or 10 μM gefitinib for 30 min before stimulation. Results represent one typical of four independent experiments.

We next examined the ability of neurotensin to induce tyrosine phosphorylation of EGFR in HCT116 cells. Figure 5A shows that treating the cells with neurotensin or EGF resulted in phosphorylation of the EGFR. Although the effect of neurotensin was clearly less than that of EGF, the phosphorylation induced by both these agonists was blocked by pretreatment with the EGFR tyrosine kinase inhibitor gefitinib (Figure 5A). Moreover, we found that neurotensin stimulated phosphorylation of Shc (Figure 5B), which is an adaptor protein that binds to, and is phosphorylated by, active RTKs [39,40]. Taken together, these results suggest that the EGFR can be transactivated by neurotensin in HCT116 cells.

Pretreatment with gefitinib strongly attenuated neurotensin-induced phosphorylation of Akt in HCT116 cells (Figure 5C). In these experiments, TGFα was used as the EGFR ligand, and the effect of TGFα on Akt phosphorylation was completely abolished by gefitinib. Neurotensin also induced Akt phosphorylation in HT29 and Panc-1 cells (Figure 4). Whereas this effect was abolished by pretreatment with gefitinib in HT29 cells (Figure 4B), neither gefitinib nor the PKC inhibitor GF109203X inhibited neurotensin-stimulated Akt phosphorylation in Panc-1 cells (Figure 4A).

### Neurotensin-induced transactivation of the EGFR is partly mediated by shedding of extracellular ligands

**Figure 6:**
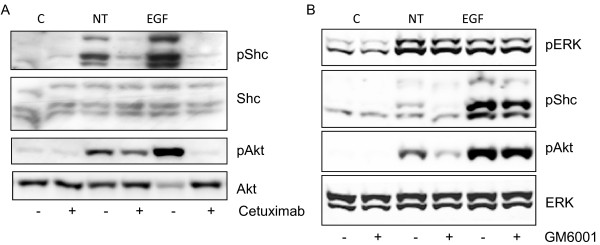
**Role of EGFR in neurotensin-induced phosphorylation of ERK, Akt, and Shc in HCT116 cells**. A, Effect of EGFR inhibition on neurotensin (NT)-induced signalling. Quiescent cells were pretreated with 25 μg/ml cetuximab for 30 min before stimulation with 1 μM NT or 10 nM EGF for five min. Cells were harvested for subsequent Western analysis as described in Methods. Results represent one typical of four independent experiments. B, Effect of protease inhibition on NT-induced phosphorylation of downstream proteins. Quiescent cells were pretreated with 10 μM GM6001 for 30 min prior to stimulation with 1 μM NT or 10 nM EGF for five min. Results represent one typical of six independent experiments.

Evidence from many cell types indicates that transactivation of the EGFR induced by GPCRs may be mediated by the activation of cell surface proteinases, resulting in subsequent shedding of EGFR ligands [41,42], or by intracellular mechanisms involving kinases such as Src and Pyk2 [43,44]. To explore further the mechanism of the gefitinib-sensitive Akt phosphorylation induced by neurotensin, we examined the effect of cetuximab, an antibody which binds to the extracellular domain of the EGFR and thereby blocks the ability of ligand-induced activation. As expected, EGF-stimulated phosphorylation of both Shc and Akt was completely inhibited by cetuximab (Figure 6A). Cetuximab pretreatment also blocked neurotensin-stimulated Shc phosphorylation, suggesting the involvement of a ligand-dependent mechanism. Neurotensin-induced phosphorylation of Akt was also inhibited by cetuximab, but only partially. We next pretreated the cells with GM6001, a broad-spectrum inhibitor of matrix and metalloproteinases (MMPs) and a disintegrin and metalloproteinases (ADAMs). Pretreatment with GM6001 did not affect the effect of neurotensin on ERK, but markedly reduced neurotensin-induced phosphorylation of Akt (Figure 6B). These results support a role of release of EGFR ligand(s) in neurotensin-stimulated phosphorylation of EGFR and Akt. However, since neither cetuximab nor GM6001 completely abolished the effect of NT on Akt phosphorylation, it seems likely that additional mechanisms are operating. As expected, the effect of exogenous EGF was insensitive to GM6001 (Figure 6B).

### Role of Ca^2+ ^in activation of PI3K/Akt

**Figure 7:**
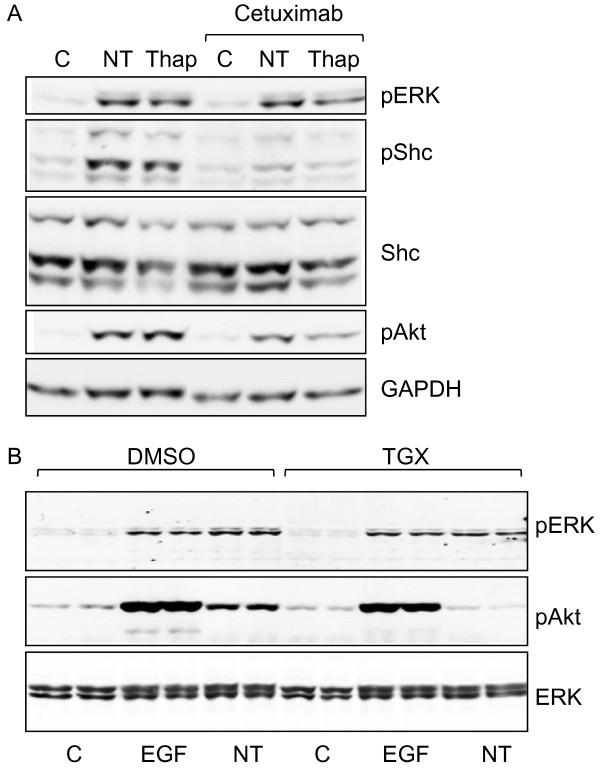
**Role of calcium in phosphorylation of ERK, Akt, and Shc**. A, Effect of thapsigargin (Thap) and neurotensin (NT) on phosphorylation of ERK, Akt, and Shc in HCT116 cells. Quiescent cells were pretreated with vehicle (0.9% NaCl) or cetuximab (25 μg/ml) for 30 min before stimulation with the vehicle (0.5% DMSO), 1 μM NT, or 1 μM Thap for five min. Cells were harvested for subsequent Western analysis as described in Methods. Results represent one typical of three independent experiments. B, Effect of PI3Kβ inhibitor on NT-stimulated ERK and Akt phosphorylation in Panc-1 cells. Quiescent cells were pretreated with vehicle (0.5% DMSO) or 1 μM TGX-221 for 30 min before stimulation with 10 nM EGF or 1 μM neurotensin (NT) for 5 min. Cells were harvested for subsequent Western analysis as described in Methods. Results represent one typical of four experiments.

The results above suggest that neurotensin-stimulated phosphorylation of Akt in HCT116 cells is mediated, at least in part, through transactivation of the EGFR. In search for mechanisms that mediate the release of EGFR ligands in HCT116 cells, we next examined the role of intracellular Ca^2+^. Thapsigargin, which increases the intracellular Ca^2+^-level by inhibiting the SERCA pump [45], induced phosphorylation of Shc, ERK and Akt (Figure 7A). Furthermore, like the effect of neurotensin, the effect of thapsigargin on Shc phosphorylation was abolished by pretreatment with cetuximab, while the effect on Akt phosphorylation was attenuated, which suggests the involvement of Ca^2+ ^in the response of the PI3K pathway to neurotensin. Further experiments showed that the effects of neurotensin and thapsigargin on Akt phosphorylation were sensitive to chelating Ca^2+^-inhibitors (data not shown). However, we have so far not been able to show that this effect is selective, as EGF-stimulated Akt phosphorylation was also attenuated by Ca^2+^-inhibitors. In contrast to the findings in HCT116 cells, thapsigargin did not stimulate phosphorylation of Akt in Panc-1 cells (data not shown). However, in these cells neurotensin-stimulated Akt phosphorylation was abolished by pretreating the cells with TGX-221, an inhibitor of PI3Kβ [46] (Figure 7B). This indicates that PI3Kβ is involved in neurotensin-induced activation of Akt in Panc-1 cells.

### Signalling pathways involved in neurotensin-induced DNA synthesis in HCT116 cells

**Figure 8:**
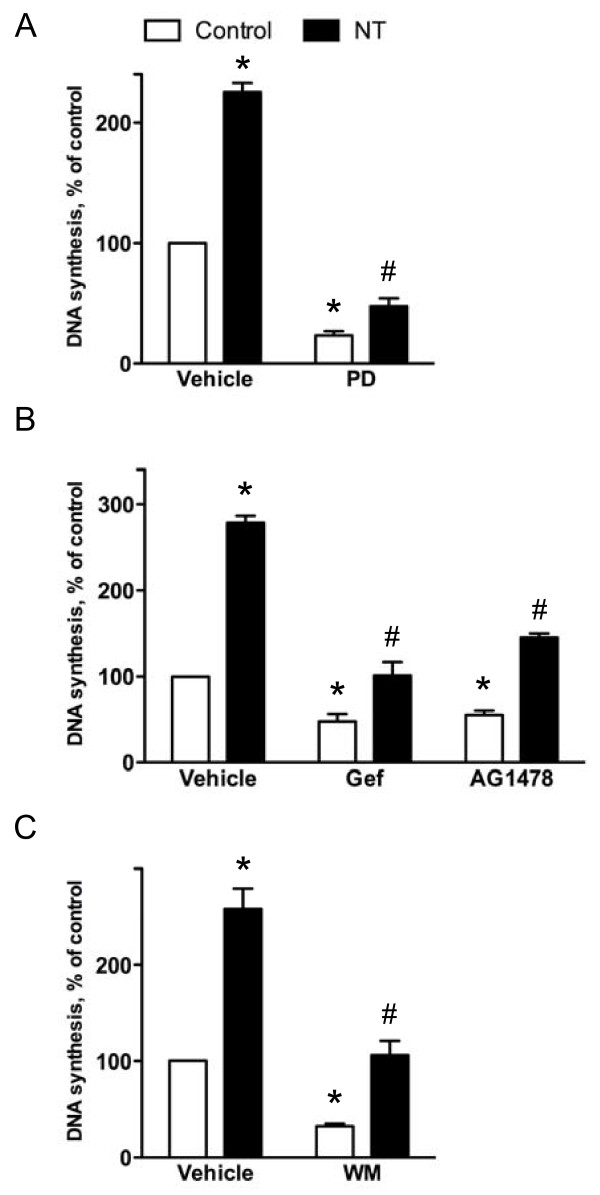
**Signalling pathways involved in neurotensin-stimulated DNA synthesis in HCT116 cells**. A, Effect of MEK inhibition on basal and neurotensin (NT)-induced DNA synthesis. Quiescent cells were pretreated with vehicle (0.5% DMSO) or 50 μM PD98059 (PD) for 30 min before stimulation with the vehicle (0.9% NaCl) or 1 μM NT. Data are presented as per cent of control values of six independent experiments. Error bars indicate S.E.M. * Significantly different from vehicle-treated control (p < 0.05), #significantly different from control cells pretreated with PD98059 (p < 0.05). B, Effect of EGFR kinase inhibitors on DNA synthesis induced by neurotensin. Quiescent cells were pretreated for 30 min with vehicle (0.5% DMSO), 10 μM gefitinib (Gef), or 10 μM AG1478 prior to stimulation with 1 μM NT. Data are presented as per cent of control values of four (AG1478) or seven (gefitinib) independent experiments. Error bars indicate S.E.M. * Significantly different from vehicle-treated control (p < 0.05), #significantly different from control cells pretreated with gefitinib or AG1478 (p < 0.05). C, Effect of phosphoinositide 3-kinase inhibition on DNA synthesis induced by neurotensin. Quiescent cells were pretreated for 30 min with vehicle (0.5% DMSO) or 1 μM wortmannin (WM) prior to stimulation with 1 μM NT. DNA synthesis was determined by [3 H]thymidine incorporation as described in the legend of figure 1. Data are presented as per cent of control values of six independent experiments. Error bars indicate S.E.M. * Significantly different from vehicle-treated control (p < 0.05), #significantly different from control cells pretreated with wortmannin (p < 0.05).

The above results suggest a role for the PLC/PKC pathway in the DNA synthesis induced by neurotensin in HCT116 cells. Furthermore, consistent with a role of ERK in the mitogenic response, pretreatment of the cells with the MEK inhibitor PD98059 (50 μM) strongly reduced both basal and neurotensin-induced DNA synthesis (Figure 8A). Although stimulation with EGF only slightly affected DNA synthesis in the cells (Figure 1), we examined the possibility that activation of the EGFR pathway might play a role in neurotensin-induced mitogenic stimulation. We found that inhibition of the EGFR tyrosine kinase activity by gefitinib or AG1478 resulted in a reduction of both basal and neurotensin-induced DNA synthesis (Figure 8B). Furthermore, a role for the PI3K pathway in the neurotensin-induced mitogenesis was likely since the DNA synthesis was reduced by the PI3K inhibitor wortmannin (Figure 8C).

## Discussion

In the present study, we have found that neurotensin-induced signalling in colon carcinoma cells involves both EGFR-dependent and -independent pathways. In HCT116 cells, stimulation by neurotensin of ERK phosphorylation and DNA synthesis is mediated by PKC, whereas Akt phosphorylation induced by neurotensin is dependent on EGFR-mediated signalling.

In agreement with previous studies in human pancreatic cancer cells (Panc-1) [23,25] we found that neurotensin-induced ERK activation and DNA synthesis in the colon cancer cells HCT116 was mainly dependent on PKC and did not involve EGFR transactivation. Thus, the stimulatory effect of neurotensin and TPA on DNA synthesis was of the same magnitude, and stimulation of both DNA synthesis and ERK phosphorylation by neurotensin was inhibited by pretreatment with the PKC blocker GF109203X. Furthermore, while neurotensin stimulated Akt phosphorylation in an EGFR-dependent manner, TPA did not induce phosphorylation of Akt in HCT116 cells. In prostate cancer cells, neurotensin also stimulated ERK phosphorylation in a PKC-dependent manner, but in these cells activation of PKC mediated transactivation of the EGFR [24].

We did not find that EGF stimulated DNA synthesis significantly in HCT116 cells. A plausible explanation is the autocrine production of TGFα and other ligands, leading to constitutive activation of EGFR in HCT116 cells [47-49]. It was previously reported that while exogenous addition of EGF had no effect on DNA synthesis, due to the production of TGFα [35], the EGFR was not saturated by the autocrine ligand(s) and could be further activated by exogenous EGF, resulting in integrin α_2 _expression, cell adhesion, and micromotion [37]. It is likely that basal DNA synthesis reflects the effect of this constitutive EGFR activation, consistent with the finding that inhibition of EGFR activity with gefitinib reduced both basal and neurotensin-stimulated DNA synthesis. However, neurotensin still enhanced DNA synthesis compared to its corresponding control.

While neurotensin-induced phosphorylation of ERK and stimulation of DNA synthesis in HCT116 cells were dependent on PKC, we found phosphorylation of Akt induced by neurotensin to be independent of PKC. Moreover, the lack of effect of TPA on phosphorylation of Akt further strengthens the notion that PKC is not involved in activation of Akt in HCT116 cells. Instead, neurotensin-induced phosphorylation of Akt was dependent on EGFR activation, and this effect was mimicked by elevation of intracellular Ca^2+ ^induced by thapsigargin. Our results thus strongly suggest that neurotensin-induced phosphorylation of ERK and Akt is mediated by different pathways. In contrast, phosphorylation of both ERK and Akt induced by neurotensin was mediated by PKC-dependent EGFR transactivation in prostate cancer cells [24]. Furthermore, in HT29 cells, both ERK and Akt phosphorylation induced by neurotensin was abolished by pretreatment with gefitinib (Figure 4) or cetuximab (data not shown). These observations are in line with previous studies in HT29 cells, demonstrating that activation of PAR1 and PAR2 receptors led to transactivation of the EGFR through matrix metalloproteinase-dependent release of TGFα [50,51]. The different time course of ERK and Akt phosphorylation in HCT116 cells also supports the involvement of different pathways.

Conflicting results have been reported on the effect of neurotensin on EGFR phosphorylation in different cells [23,24]. Thus, while neurotensin did not induce transactivation of the EGFR in Panc-1 cells [23], PKC-dependent transactivation of the EGFR mediated the mitogenic effect of neurotensin on prostate cancer cells [24]. We found that neurotensin induced phosphorylation of the EGFR and the adaptor protein Shc in HCT116 cells, and that inhibiting the EGFR with cetuximab or gefitinib strongly reduced neurotensin-induced phosphorylation of Akt. These results strongly suggest that the EGFR is transactivated by neurotensin in HCT116 cells and that this transactivation is involved in mediating the Akt phosphorylation stimulated by neurotensin. Since the PI3K/Akt pathway is important in several regulations besides cell proliferation, such as promoting cell survival by enhancing resistance to apoptosis [52-56], the EGFR-mediated activation by neurotensin may have significant roles in the malignant phenotype in these cells.

It is unclear why neurotensin activates different pathways in the different the cell lines. It is known that HCT116 and Panc-1 cells both harbour a *KRAS *mutation, while HT29 cells have a mutant *BRAF*. Furthermore, HT29 and HCT116 cells harbour mutations in the catalytic α polypeptide of phosphoinositide-3-kinase, (*PIK3CA*), and HT29 cells also have mutated p53 http://www.sanger.ac.uk/genetics/CGP/CellLines/, [57]. While it is known that mutations in *KRAS*, *BRAF *and *PIK3CA *may determine the responsiveness to targeted therapies such as EGFR, MEK or mTOR inhibitors [53,57,58], the consequences of these mutations for neurotensin signalling in the different cell lines are not obvious. Whereas we found that neurotensin treatment stimulated Akt phosphorylation in the three cell lines examined, an earlier report using NTSR1-transfected AV12 cells found that neurotensin inhibited basal and EGF- or insulin-stimulated Akt phosphorylation, which was ascribed to a negative regulation mediated through Gq [59]. It has been found that classical PKC isoforms mediate feedback inhibition of EGFR transactivation by Gq-coupled receptor agonists [60]. The degree of EGFR-induced transactivation involvement in signalling by neurotensin may thus depend on the strength of PKC-mediated feedback inhibition in different cells. In this context, it is of interest that HCT116 cells have a higher expression of the classical isoform PKCβII than HT29 cells [61].

Interestingly, while the results showed that EGFR activation was required for neurotensin-stimulated phosphorylation of Akt, we did not obtain complete inhibition of this effect by pretreatment with neither GM6001, cetuximab or gefitinib. Contrary to this, Akt phosphorylation induced by direct activation of the EGFR by TGFα or EGF was completely suppressed by gefitinib or cetuximab. Also, neurotensin-stimulated Shc phosphorylation was completely suppressed. One possible explanation is that neurotensin also might induce release of ligands that activate ErbB3 or ErbB4 receptors. The HCT116 cells have been found to release several ligands that activate the ErbB receptor family [62-64]. The lack of complete inhibition induced by GM6001 pretreatment could imply that EGFR transactivation could also be induced independently of ligand shedding by an intracellular calcium-mediated mechanism, possibly involving Pyk2 or Src [43,44]. Alternatively, neurotensin might induce transactivation of the insulin-like growth factor-1 receptor (IGF-1R), as observed in human colonic epithelial cells [65]. Another possibility is that neurotensin induces Akt phosphorylation through activation of subtypes of PI3K that are directly activated by GPCRs [66,67]. In fact, HCT116 cells have been found to express PI3Kβ [52], which is activated by GPCRs [66]. TGX-221, an inhibitor of PI3Kβ [46], did not affect neurotensin-stimulated Akt phosphorylation when used alone, but it further suppressed neurotensin-stimulated phosphorylation of Akt when combined with gefitinib (data not shown). Thus, it is possible that multiple pathways activated by neurotensin might converge on Akt phosphorylation in a partially redundant manner. In contrast, neurotensin-stimulated phosphorylation of Akt in Panc-1 cells was abolished by pretreatment with TGX-221, indicating involvement of PI3Kβ in this cell line. Although several mechanisms may thus be involved in mediating the effect of neurotensin on phosphorylation of Akt in HCT116 cells, our results suggest that ligand shedding, which may be dependent on Ca^2+ ^elevation, and the resulting activation of the EGFR is a main pathway.

## Conclusions

**Figure 9:**
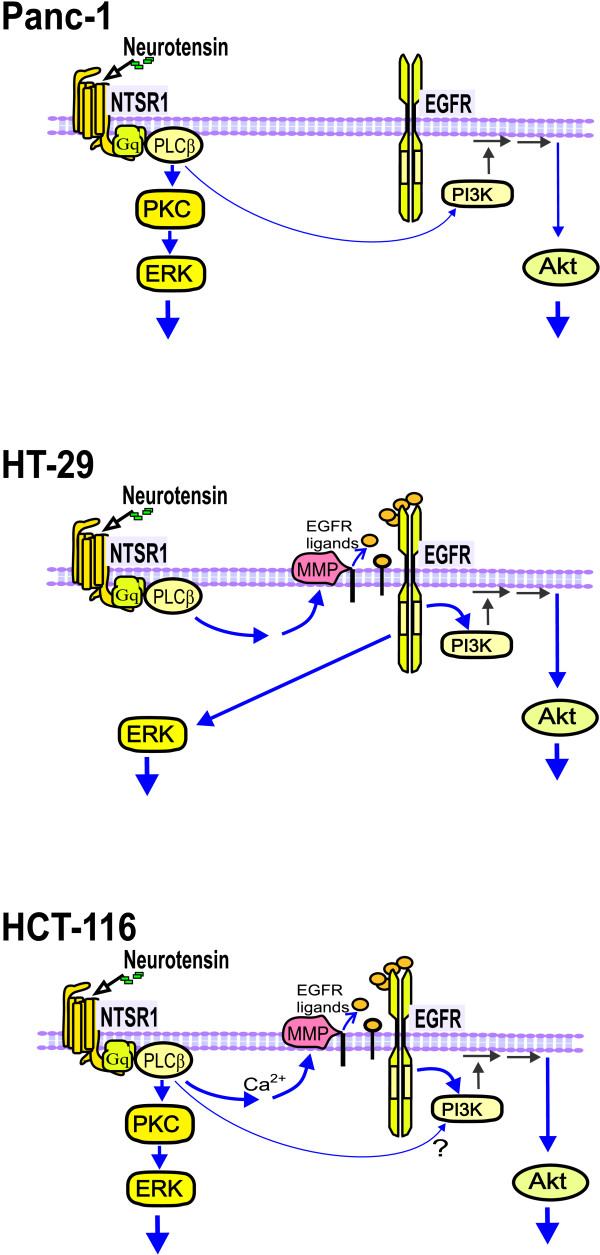
**Summary of pathways activated by neurotensin in HCT116, HT29, and Panc-1 cells**.

While acting predominantly through PKC in Panc-1 cells and via EGFR transactivation in HT29 cells, neurotensin used both these pathways in HCT116 cells (Figure 9). Taken together, our results suggest that, in HCT116 cells, neurotensin-induced DNA synthesis and phosphorylation of ERK is mediated mainly by PKC independently of EGFR transactivation. In addition, neurotensin induces phosphorylation of Akt via activation of metalloproteinases and subsequent shedding of ligands that activate the EGFR.

## List of abbreviations

ADAM: a disintegrin and metalloproteinase; EGF: epidermal growth factor; EGFR: epidermal growth factor receptor; ERK: extracellular signal-regulated kinase; GPCR: G protein-coupled receptor; MEK: mitogen-activated protein kinase kinase; MMP: matrix metalloproteinase; NF-κB: nuclear factor κB; NHE1: sodium-proton exchanger 1; PI3K: phosphoinositide 3-kinase; PIK3CA: phosphoinositide 3-kinase, catalytic α polypeptide; PKC: protein kinase C; PKD: protein kinase D; PLC: phospholipase C; S.E.M.: standard error of the mean; RTK: receptor tyrosine kinase; TCA: trichloroacetic acid; TPA: tetradecanoylphorbol-13-acetate.

## Competing interests

The authors declare that they have no competing interests.

## Authors' contributions

KMM participated in the design of the study, carried out DNA synthesis and immunoblotting experiments, and drafted the manuscript. IHT, MA, and JØ carried out immunoblotting experiments and helped revise the manuscript. MD carried out DNA synthesis and inositol phosphate experiments. OD and TC conceived of the study, participated in the design of the study and helped revise the manuscript. DS conceived of the study, participated in the design of the study, carried out DNA synthesis and inositol phosphate experiments, and helped revise the manuscript. All authors read and approved of the final manuscript.

## Pre-publication history

The pre-publication history for this paper can be accessed here:

http://www.biomedcentral.com/1471-2407/11/421/prepub
